# Advances in Marine Microbial Symbionts in the China Sea and Related Pharmaceutical Metabolites

**DOI:** 10.3390/md7020113

**Published:** 2009-04-20

**Authors:** Zhiyong Li

**Affiliations:** Laboratory of Marine Biotechnology, School of Life Sciences and Biotechnology and Key Laboratory of Microbial Metabolism, Ministry of Education, Shanghai Jiao Tong University, 800 Dongchuan Road, Shanghai 200240, P.R. China; E-mail: zyli@sjtu.edu.cn; Tel.: (+86)21-34204036

**Keywords:** Marine microbial symbionts, diversity, biological activity, natural products, gene

## Abstract

Marine animals and plants such as sponges, sea squirts, corals, worms and algae host diverse and abundant symbiotic microorganisms. Marine microbial symbionts are possible the true producers or take part in the biosynthesis of some bioactive marine natural products isolated from the marine organism hosts. Investigation of the pharmaceutical metabolites may reveal the biosynthesis mechanisms of related natural products and solve the current problem of supply limitation in marine drug development. This paper reviews the advances in diversity revelation, biological activity and related pharmaceutical metabolites, and functional genes of marine microbial symbionts from the China Sea.

## 1. Introduction

The term symbiosis was first defined by Heinrich Anton de Bary in 1879 as “he living together of unlike organisms”[1]. At present, symbiosis commonly describes close and often long-term interactions, including mutualistic, parasitic and commensal relationships, between different biological species. The mutualistic relationship (mutualism), the true symbiosis, is obligate, which means the survival of one species requires involvement of another, whereas, parasitism and commensalism are facultative. Because most symbiotic microorganisms presently remain unidentified and few true symbiotic relationships (mutualism) between hosts and microbes have been confirmed, according to the definition of Anton de Bary, the symbiotic microorganisms discussed in this review include mutualistic, parasitic and commensal microorganisms in/on marine organisms, whereas pathogenic microorganisms are excluded.

The development of marine organisms-derived compounds into drugs has been held back by supply limitations. Symbioses between microorganisms and marine organisms are abundant and widespread in the sea. Most marine invertebrates and algae harbor diverse microbial symbionts including prokaryotic bacteria, archaea, cyanobacteria, and fungi. Increasing evidence implicates microbial symbionts as the true source of many marine organism-derived compounds, which makes marine microbial symbionts a hotspot in the field of marine microbiology and marine natural products because of their potential for solving the bottleneck problem of marine natural product supply.

In this paper, the advances in diversity revelation, biological activity and related pharmaceutical metabolites, functional gene of marine microbial symbionts of marine organisms such as sponges, corals, sea squirts, holothurians, and algae in the China Sea are reviewed.

## 2. Diversity revelation of marine microbial symbionts in the China Sea

Both culture-dependent and culture-independent methods have been used to reveal the diversity of marine microbial symbionts in the China Sea. As shown in Table 1, the present research has mainly focused on sponges, and the revealed microbes mainly include actinomycetes and bacteria. Particularly, some uncultured bacterial symbionts have been observed by molecular methods. The symbiotic actinomycetes mainly consist of genus *Streptomyces*, and the revealed marine bacterial symbionts mainly consist of Proteobacteria, Bacteroidetes and Firmicutes. Fungal symbionts of the phyla Ascomycola and Basidiomycota have been observed, as well as archaeal symbiont *Cenarchaeum*.

Sponges (phylum *Porifera*) are among the oldest metazoan animals. The special two-layer structure of outer and inner endosome and special strategy for sequestering food by filtering seawater make sponges an ideal habitat for symbiotic microorganisms. In general, for high-microbial-abundance sponge, microorganisms can contribute up to 40–70% of the sponge body volume exceeding microorganisms in seawater by 2–4 orders of magnitude. Marine sponge is known as an important source for marine drug development, and accumulated evidence suggests that symbiotic microorganisms could be the true source of at least some of the biologically active metabolites isolated from sponges. So it is reasonable that the researches of marine microbial symbionts in China mainly focus on sponge microbial symbionts. Microbial symbionts of South China Sea sponges are mainly involved according to the present reports.

It is known that most of the microorganisms in nature are still uncultured in laboratory, so culture-independent molecular approach is useful to reveal the complex microbial community of marine microbial symbionts. In 2006, Li *et al*. [7] first reported the *in vivo* symbiotic microbial community of four South China Sea sponges *Stelletta tenu is*, *Halichondria rugosa, Dysidea avara*, and *Craniella australiensis* by culture-independent 16S rDNA-DGGE fingerprinting and phylogenetic analysis. The sponge *C. australiensis* was proven to have the greatest symbiotic microbial diversity, including the phyla Proteobacteria, Bacteroidetes, Firmicutes and Actinobacteria, followed by the sponge *D. avara* with the two phyla Proteobacteria and Bacteroidetes, and the sponges *S. tenuis* and *H. rugosa* with the phylum Proteobacteria. Proteobacteria, including α, β, δ subdivisions, were found to make up the majority of the predominant bacteria in these sponges. Li *et al.* [8,11] revealed the symbiotic microbial diversity of these four South China Sea sponges using 16S rDNA clone library alongside restriction fragment length polymorphism (RFLP) and phylogenetic analysis. A wide symbiotic microbial diversity was detected, abundant clones with low identify with sequences retrieved from database were found as well as uncultured sponge microbial symbionts. For instance, the *C. austrialiensis*-associated microbial community consists of α-, β-, and γ-Proteobacteria, Firmicutes, Bacteroidetes as well as Actinobacterium. The phylogenetic analysis showed that the bacterial community structure of *Stelletta tenuis* was similar to that of *Halichondria rugosa* comprising γ-Proteobacteria and Firmicutes, while, α-, γ-Protebacteria, Bacteroidetes and uncultured sponge symbionts were found in sponge *Dysidea avara*. Accordingly, a specific sponge–microbe association was suggested based on the difference of symbiotic bacterial diversity among these four sponges from the same geography location. Besides actinomycetes and bacteria, recently, *Cenarchaeum s* ymbionts have been also found in sponge *Theonella swinhoei* in the South China Sea using 16S rDNA clone library and phylogenetic analysis in the author’s laboratory.

In the case of the diversity of culturable sponge microbial symbionts, Li *et al*. [8] isolated 23 actinomycetes from sponge *Craniella australiensis* using seawater medium with sponge extracts, among which 11 isolates closely matched genus *Streptomyces*. As for sponges, *Stelletta tenuis*, *Halichondria rugosa* and *Dysidea avara*, Proteobacteria and Firmcutes represent the main cultured bacterial species of sponge bacterial symbionts [12]. *Streptomyces*, *Pseudonocardia* and *Nocardia* were isolated from the four South China Sea sponges *Craniella australiensis*, *Halichondria rugosa*, *Sponge* sp. and *Stelletta tenuis* [9]. Jiang *et al*. [6] described actinobacteria isolated from the marine sponge *Haliclona* sp. collected in shallow water of the South China Sea. A total of 54 actinobacteria were isolated using media selective for actinobacteria. The phylogenetic analysis based on 16S rRNA gene sequencing showed that the isolates belonged to the genera *Streptomyces*, *Nocardiopsis*, *Micromonospora* and *Verrucosispora*. In addition, Jiang *et al*. [5] studied the diversity of actinobacteria isolated from the marine sponge *Iotrochota* sp. in the South China Sea. The phylogenetic analysis based on 16S rRNA gene sequencing showed that the isolates belonged to genera *Streptomyces*, *Cellulosimicrobium*, and *Nocardiopsis*. The majority of the isolated strains also belonged to the genus *Streptomyces* as sponge *Haliclona* sp. At present, the reports on marine fungal symbionts are very rare. In the author’s laboratory, diverse culturable fungi of the phyla Ascomycola and Basidiomycota associated with South China Sea sponges, *Phakellia fusca* and *Theonella swinhoei*, have been detected recently, which expends our knowledge of sponge symbionts.

The investigations of microbial symbionts of sponges in other China Sea areas were first found in 2006. For the sponge *Mycale adhaerens* found in the sea near Hong Kong, a highly stable and distinctive bacteria–sponge association irrespective of the environmental conditions, mainly Proteobacteria, was revealed by Lee *et al*. [14], suggesting that the associations between sponge and bacteria are consistent and specific. Qian *et al*. [15] compared the microbial communities of the sponge *Callyspongia* sp. from Hong Kong and the sponge *Callyspongia plicifera* from the Bahamas, and suggested that the two congeneric sponges *Callyspongia* spp. from different biogeographic regions have different bacterial associates, where the bacterial communities of sponge *Callyspongia* sp. from Hong Kong, including Proteobacteria, Firmicutes, Bacteroides and Actinobacteria, were more diverse. From the sponge *Hymeniacidon perleve* in the Yellow Sea, actinobacteria were isolated by Zhang *et al*. [2,4]. A phylogenetic analysis using 16S rRNA gene sequences revealed that the isolates belonged to seven genera of culturable actinobacteria including *Actinoalloteichus*, *Micromonospora*, *Nocardia*, *Nocardiopsis*, *Pseudonocardia*, *Rhodococcus*, and *Streptomyces*. Like the South China Sea sponges, the dominant genus was *Streptomyces*. Using culture-independent molecular method, Xin *et al*. [3] revealed eight actinobacterial genera: *Acidimicrobium*, *Actinomyces*, *Corynebacterium*, *Propionibacterium*, *Micrococcus*, *Microbacterium*, *Streptomyces*, *Sporichthya* and unidentified actinobacterial clones in Yellow Sea sponge *Hymeniacidon perleve*.

All the above reports of cultured sponge microbial symbionts are based on cultivation of single pure isolates. In 2007, Li *et al*. [13] reported the culturable symbiotic bacteria diversity in mixed cultures of four South China Sea sponges *Stelletta tenuis*, *Halichondria rugosa*, *Dysidea avara*, and *Craniella australiensis* using PCR-DGGE fingerprinting and 16S rDNA phylogenetic analysis. Diverse bacteria such as α-, γ- and δ-Proteobacteria, Bacteroidetes and Firmicutes were detected in the mixed cultures, some of which were previously uncultivable bacteria, potential novel strains with less than 95% similarity to their closest relatives and sponge symbionts growing only in the medium with sponge extract. The composition of the cultivable bacterial community in the mixed culture was different, depending on the medium and sponge species. Sponge *S. tenuis* was found to have the highest cultivable bacterial diversity including α-,γ- and δ-Proteobacteria, Bacteroidetes, and Firmicutes, followed by sponge *Dysidea avara* without δ-Proteobacteria, sponge *Halichondria rugosa* with only α-, gamma;-Proteobacteria and Bacteroidetes, and sponge *C. australiensis* with only α-,γ-Proteobacteria and Firmicutes.

Compared to sponge microbial symbionts, reports of other marine organisms are rarely found (Table 1). *Micromonospora* sp, and *Streptomyces* sp. were isolated from the sea hare *Aplysia dactylomela* and the marine alga *Gracilaria verrucosa* [10]. Zheng *et al*. [16] isolated diverse bacteria including *Alteromonas* sp*, Bacillus* sp*., Flavobacterium* sp. and *Pseudomonas* sp. from the coral *Eupexaura currata*, and seaweeds such as *Gymnogongrus flabelliformis, Lomentaria catenata, Laurencia flabelliformis, Laminaria japonica, Ulva fasciata, Ulva pertusa,* and *Sargassum thunbergii.* Dobretsov *et al*. [17] and Harder *et al*. [18] detected three major groups of bacteria: α- and γ-Proteobacteria and Cytophaga–Flexibacter–Bacterioides on the surface of the soft coral *Dendronephthya* sp. found in Hong Kong.

## 3. Biological activity and pharmaceutical metabolites of marine microbial symbionts in the China Sea

Since 2000, investigations of isolation and bioassay of marine microorganisms associated with marine organisms in the China Sea have been made. Antimicrobial activity has been observed for most of the marine microbial symbionts. For example, 20.6% of marine actinomycetea, which were isolated from sea plants and animals such as sea hare, *Aplysia dactylomela*, sea anemone, *Actiniaria*, and marine plants, *Ulva lactuca* and *Gracilaria verrucosa* in the intertidal zone of Xiamen Island, displayed cytotoxic activity on P388 cells and 18.6% on KB cells [10]. 20% of bacteria from marine sponge and coral and 11% of bacteria from seaweeds in different coastal areas of the China Sea showed positive antimicrobial activity [16]. In the study of Li *et al*. [12], a total of 399 bacteria were isolated from the sponges *S. tenuis*, *H. rugosa*, and *D. avar*a in the South China Sea, among which, 13 isolates from *S. tenuis*, 42 from *H. rugosa*, and 20 from *D. avara* showed pronounced broad-spectrum antimicrobial activities and enzymatic potentials. Similarly, actinomycetes isolated from sponge *C. austrialiensis* were proved to have a broad-spectrum of antimicrobial activity [8]. Twenty-nine marine bacterial strains were isolated from the sponge *Hymeniacidon perleve* at Nanji Island, and antimicrobial screening showed that eight strains inhibited the growth of terrestrial microorganisms [20].

At present, the researches of pharmaceutical metabolites of marine microbial symbionts in the China Sea mainly focus on sponge microbial symbionts and fungal symbionts are mainly involved (Table 2).

The major antimicrobial metabolite isolated from *Pseudoalteromonas piscicida* NJ6-3-1 with wide antimicrobial spectrum was identified as norharman (**1**) (a beta-carboline alkaloid) [20]. Cyclo-(L-Pro- L-Phe) (**2**) was isolated from *Alcaligenes faecalis* A72 associated with the sponge *S. tenuis* showed moderate inhibitory activity against *S. aureus* [21]. Bioassay-guided fractionation of the CHCl_3_ extract of the fermentation broth of a sponge *Mycale plumose*-derived actinomycete *Saccharopolyspora* sp. nov., led to the isolation of two known prodigiosin analogs – metacycloprodigiosin (**3**) and undecylprodigiosin (**4**). These compounds exhibited significant cytotoxic activities against five cancer cell lines: P388, HL60, A-549, BEL-7402 and SPCA4 [22]. Bacillamides have been proved to inhibit the growth of red-tide algae such as *Cochlodinium polykrikoides* [23]. A novel thiazole alkaloid, neobacillamide A (**5**), together with a known related one, bacillamide C (**6**), were isolated from *Bacillus vallismortis* C89 associated with the South China Sea sponge *Dysidea avara* [24]. A new compound, (*S*)-2,4-dihydroxy-1-butyl(4-hydroxy) benzoate (**7**), and a known compound, fructigenines A (**8**) exhibiting cytotoxic activity against tsFT210 cells, were isolated from fungus *Penicillium auratiogriseum* derived from sponge *Mycale plumose* [25]. Xie *et al*. [26] demonstrated that two antifungal trichothecenes, including roridin A (**9**) and roridin D (**10**) produced by the fungus *Myrothecium* sp. isolated from the marine sponge *Axinella* sp. in the South China Sea, could be potential inhibitors against the plant pathogen *S. sclerotiorum*. In 2007, Yang *et al*. [27] reported that the sponge *Acanthella cavernosa*-associated fungus, *Letendraea helminthicola*, produced two antifouling compounds: 3-methyl-*N*-(2-phenylethyl) butanamide (**11**) and cyclo(D-Pro-D-Phe) (**12**). Three new quinazoline alkaloids, aurantiomides A, B, and C were isolated from *Penicillium aurantiogriseum* SP0-19 associated with sponge *Mycale plumose* collected in Jiaozhou Bay, Qingdao, P.R. China [28], among which aurantiomides B (**13**) and C (**14**) showed moderate cytotoxicities against HL-60, P388 and BEL-7402, P388 cell lines, respectively.

Besides sponges, some pharmaceutical metabolites have been isolated from marine microbial symbionts associated with sea squirts, marine bryozoans, molluscs and algae (Table 2). 5α,8α-epidioxy-23-methyl-(22*E*,24*R*)-ergosta-6,22-dien-3β-ol (**15**) with cytotoxic activity was isolated from the fungus *P. stoloniferum* QY2-10, associated with a sea squirt collected in Jiaozhou Bay, Qingdao, P.R. China [29]. Six new ergosterols were obtained from a marine *Bugula* sp. derived fungus *Rhizopus* sp. along with four known ones; these compounds **16–25** showed activities against P388 and HL-60, A549 and BEL-7402. [30]. Seven new prenylated indole diketopiperazine alkaloids, including spirotryprostatins C–E (**26**), 2 derivatives of fumitremorgin B (**27**), and 13-oxoverruculogen (**28**), have been isolated from the holothurian-derived fungus *Aspergillus fumigatus*. These compounds showed activity to four cancer cell lines MOLT-4, A549, HL-60, and BEL-7420 cell lines [31]. Lasiodiplodin (**29**), which could inhibit the *in vitro* growth of *S. aureus*, *Bacillus subtilis*, and *Fusarium oxysporum,* were isolated from the mycelium extracts of a brown alga endophytic fungus (No. ZZF36) obtained from the Zhanjiang sea area by Yang *et al*. [32]. A new naphthoquinoneimine derivative, 5,7-dihydroxy-2-[1-(4-methoxy-6-oxo-6H-pyran-2-yl)-2-phenylethylamino]-[1,4]-naphthoquinone (**30**), and asperamides A (**31**), nigerasperone C (**32**) from the marine brown alga *Colpomenia sinuosa-*derived endophytic fungus *Aspergillus niger* EN-13 displayed moderate activity against *Candida albicans* [33–35]. Chaetopyranin (**33**) isolated from *Chaetomium globosum*, an endophytic fungus derived from the marine red alga *Polysiphonia urceolata*, exhibited moderate to weak cytotoxic activity [36].

Compared with the small molecular metabolites of marine microbial symbionts, the investigation of pharmaceutical big molecule metabolites is rarely involved. The gene cloning, purification, properties, kinetics, and antifungal activity of chitinase from marine *Streptomyces* sp. DA11 associated with the South China Sea sponge *Craniella australiensis* were investigated by Han *et al*. [37]. Antifungal activities were observed against *Aspergillus niger* and *Candida albicans*, suggesting the potential as an antifungal agent.

In addition to the isolation of pharmaceutical metabolites, the optimized production of metabolites has been carried out. For example, to optimize the production of the antifouling compounds 3-methyl-*N*-(2-phenylethyl) butanamide and cyclo (D-Pro-D-Phe), Yang *et al*. [27] examined the production of compounds under different culture conditions (temperature, salinity, pH, and carbon and nitrogen sources). The results indicated that culture conditions greatly affected the production of bioactive compounds and that the conditions favorable for fungal growth might not be the best conditions for bioactive compound production. Han *et al*. [38] used statistical Plackett–Burman design and Box–Behnken Response Surface Methodology to optimize the medium components to improve the chitinase activity of *Streptomyces* sp. DA11 associated with the South China Sea sponge *Craniella australiensis*. As a result, with the optimized medium, both the chitinase activity and cell growth were remarkably enhanced.

## 4. Screening of functional gene from marine microbial symbionts in the China Sea

The study of functional genes of marine microbial symbionts has been started in China recently. For instance, nonribosomal peptide synthetase (NRPS) adenylation (A) domain genes in bacteria isolated from four South China Sea sponges, *Stelletta tenuis*, *Halichondria rugosa*, *Dysidea avara* and *Craniella australiensis* were investigated by Zhang *et al*. [39]. Meanwhile, the antimicrobial bioassays of bacteria with NRPS genes were carried out to confirm the screening of NRPS genes. As a result, fifteen bacteria grouped into two phyla Firmicutes (13 of 15) and Proteobacteria (two of 15) according to 16S rDNA sequences were found to contain NRPS genes. Based on the phylogenetic analysis of the conserved A domain amino acid sequences, most of the NRPS fragments (11 of 15) showed below 70% similarity to their closest relatives suggesting the novelty of these NRPS genes. All of the 15 bacteria with NRPS genes have antimicrobial activities, most of them exhibited activity against multiple indicators including fungi and gram-positive and gram negative bacteria. The different antimicrobial spectra indicated the chemical diversity of biologically active metabolites and the possible role of bacterial symbionts in the host’ antimicrobial chemical defense. Meanwhile, eighteen bacteria with KS (ketosynthase) genes were identified by PCR screening of 98 isolates from these four sponges by Zhang *et al*. [40]. All the KS domains were found to belong to trans-AT type I PKSs and matched PKSs of marine bacterial symbionts. The 18 bacteria exhibited broad-spectrum antimicrobial activities against fungi, gram-positive and gram-negative bacteria. A 21.8-kb PKS gene cluster fragment containing five modules was isolated from *Staphylococcus lentus* A75 associated with sponge *Stelletta tenuis* by screening of a fosmid library. The PKS gene diversity and different antimicrobial spectra indicated the potential of bacterial symbionts of South China Sea sponges for diverse polyketides production.

PKS and NRPS sequences were detected in more than half of the actinobacteria, including *Streptomyces*, *Cellulosimicrobium* and *Nocardiopsis*, isolated from the marine sponge *Iotrochota* sp. in the South China Sea [5]. As for 24 actinobacteria isolated from marine sponge *Haliclona* sp. in the South China Sea, PKS and NRPS sequences were detected in more than half of the isolates and the different “PKS-I—PKS-II—NRPS” combinations in different isolates belonging to the same species were indicators of their potential natural product diversity and divergent genetic evolution [6].

Traditionally, the screening of useful microbial strain is mainly based on bioassays, which are restricted by the screening models used and the culture conditions resulting in the lack of related metabolites production. According to the reports above [5,6,39,40], compared with the traditional method based on bioassay only, the combined strategy of gene with bioassay provides access to finding microorganisms with the potential to efficiently synthesize bioactive compounds. The screening of metabolites related genes such as PKS, NRPS provides a direct biodiscovery for detecting the production potential of related natural products. Meanwhile, genomics-based approaches can reveal insights into the metabolic and physiological properties. As for the cultured symbionts, the identification of natural product biosynthetic genes will guide the isolation of target compounds and improve the yield of natural products in fermentation by metabolism control. For uncultured marine microbial symbionts, gene-based approach is able to investigate the potential for producing pharmaceutical natural products and the related biosynthesis pathway, consequently, related bioactive natural products can be produced by heterologous expression. In addition, metabolite related gene information based on metagenomes will guide the location of unculturable symbiont producer in the host and provide direct proof for the hypothesis of symbionts origin of second metabolites isolated from marine invertebrates.

## 5. Concluding remarks and perspectives

According to the discussion above, diverse microbial symbionts in the China Sea including actinomycetes, bacteria, archaea and fungus have been revealed by both culture-dependent and culture-independent approaches. Some progresses in biological evaluation of strains, isolation of natural products with pharmaceutical potentials, and screening of functional genes from marine microbial symbionts have been made since 2000. Even so, the study on marine microbial symbionts in the China Sea is just beginning. For instance, only few sponge species have been involved, and most of which are from the South China Sea; only a small part of isolated microorganisms, mainly fungi, have been investigated for metabolites production. Up till now, no reports on the biosynthetic pathways of pharmaceutical compounds and large-scale production have been found. Meanwhile, study on uncultured microbial symbionts has been rarely done. As mention above, mutualism represents the true symbiosis compared with parasitism and commensalism. But, no true symbionts have been confirmed because of technical limitations. Thus, the investigation of marine microbial symbionts should be expended and strengthened in China.

In the case of marine symbiotic microbial diversity and pharmaceutical metabolites, the following areas are recommended to be strengthened in future: (1) culture-independent molecular strategy and metagenomes approach for the revelation of *in vivo* microbial diversity, especially true symbionts which can be obtained by vertical transmission between generations; (2) the development of novel isolation strategy imitating the natural environment conditions in order to isolate novel symbionts especially the uncultured symbionts; (3) effective isolation technique for trace natural products and high-flux screening technique for the assay of biological activity; (4) screening of gene cluster involved in the biosynthesis of pharmaceutical metabolites and revelation of biosynthesis mechanisms; (5) heterologous expression of pharmaceutical metabolites of uncultured symbionts and large-scale production of natural products by large-scale fermentation.

## Figures and Tables

**Table 1:** The diversity of marine microbial symbionts in the China Sea

Host	Microorganism	Ref.
	**Actinomycetes**	
Sponge *Hymeniacidon perleve*	*Actinoalloteichus cyanogriseus, Actinoalloteichus hymeniacidonis* sp. nov., *Acidimicrobium, Actinomyces, Corynebacterium, Micromonospora aurantiaca, Micrococcus* sp., *microbacterium, Nocardiopsis dassonvillei, Nocardiopsis lucentensis, Nocardia salmonicida, Rhodococcus opacus, Propionibacterium, Pseudonocardia antarctica, Sporichthya* sp., *Streptomyces argenteolus, Streptomyces aureofaciens, Streptomyces caviscabies, Streptomyces coelicolor, Streptomyces gibsonii, Streptomyces gougerotii, Streptomyces paradoxus, Streptomyces rimosus, Streptomyces tendae*	[2,3,4]
Sponge *lotrochota* sp.	*Cellulosimicrobium cellulans, Nocardiopsis, Streptomyces bikiniensis, Streptomyces fradiae, Streptomyces geysiriensis, Streptomyces griseoflavus, Streptomyces griseus* subsp., *Streptomyces intermedius, Streptomyces sindenensis, Streptomyces parvus, Streptomyces variabilis*	[5]
Sponge *Halidona* sp.	*Micromonospora carbonacea, Micromonospora floridensis, Micromonospora* sp., *Nocardiopsis* sp., *Streptomyces fradiae, Streptomyces griseoincarnatus, Streptomyces variabilis, Verrucosispora gifhornensis*	[6]
Sponge *Craniella australiensis*	Actinomycetales bacterium, *Streptomyces* sp., *Pseudonocardia* sp., uncultured actinobacterium	[7, 8, 9]
Sponges *Stelletta tenuis, Halichondria rugosa, Reniochalina* sp.	*Streptomyces* sp., *Pseudonocardia* sp.	[9]
Sponge *Sponge* sp.	*Acidimicrobium, Cellulosimicrobium, Mycobacterium, Nocardia* sp., *Streptomyces* sp., *Pseudonocardia* sp.	[3,9]
Sea hare *Aplysia dactylomela,* marine alga *Gracilaria verrucosa*	*Micromonospora* sp., *Streptomyces* sp.	[10]
	**Bacteria**	
Sponge *Stelletta tenuis*	*Adelie penguin guano bacterium, Alcaligenes faecalis, Acinetobacter johnsonii, Bacillus cereus, Bacillus firmus, Bacillus sporothermodurans, Bdellovibrio* sp., *Brevundimonas vesicularis, Halomonas* sp., *Idiomarina* sp., *Marinomonas* sp., *Oceanisphaera litoralis, Oleiphilus messinensis, Psychrobacter glacincola, Psychrobacter maritimus, Psychrobacter psychrophilus, Psychrobacter luti, Shewanella pacifica, Sporosarcina* sp., *Staphylococcus epidermidis, Staphylococcus lentus, Vitellibacter vladivostokensis,* uncultured Arctic sea ice bacterium, uncultured Antarctic sea ice bacterium, uncultured alpha-Protebacterium, uncultured bacterium, uncultured *Clostridia* bacterium	[7, 11, 12, 13]
Sponge *Halichondria rugosa*	*Acinetobacter johnsonii, Acidovorax* sp., *Alcaligenes* sp., *Bacillus anthracis, Bacillus licheniformis, Bacillus psychrophilus, Bacillus subtilis, Brevundimonas vesicularis, Halomonas* sp., *Idiomarina* sp. NT, *Marinomonas* sp., *Moellerella wisconsensis, Psychrobacter maritimus, Psychrobacter psychrophilus, Pseudomonas* sp., *Paracoccus halodenitrificans, Providencia* sp., *Rhodobacteraceae bacterium, Stenotrophomonas maltophilia,* marine bacterium KMM 3937, rainbow trout intestinal bacterium, uncultured gamma-Proteobacterium, uncultured alpha-Proteobacterium, uncultured bacterium	[7, 11, 12, 13]
Sponge *Dysidea avara*	*Acidovorax* sp., *Alcaligenes* sp., *Arenibacter latericius, Bellia baltica, Bacillus cereus, Biziolla paragoriae, Bizionia paragorgiae, Bacillus vallismortis, Brevundimonas vesicularis, Erythobacter lutedus, Flavobacteriaceae* str., *Halomonas* sp., *Idiomarina* sp., *Klebsiella pneumoniae, Marinobacter* sp., *Oceanisphaera koreensis, Oceanisphaera* sp., *Psychrobacter cibarius, Psychrobacter fozii, Psychrobacter cibarius, Psychrobacter psychrophilus, Psychrobacter* sp., *Paracoccus* sp., *Rhizobiaceae bacterium, Staphylococcus epidermidis,* uncultured sponge symbiont, uncultured bacteroidetes bacterium, uncultured Arctic sea ice bacterium, uncultured *Pseudoalteromonas* sp., uncultured proteobacterium	[7, 11, 12, 13]
Sponge *Craniella australiensis*	*Aequorivitaferruginea, Alcaligenes* sp., *Bacillus* sp., *Bizionia paragorgiae, Brevundimonas vesicularis, Carnobacterium funditum, Cytophaga* sp., *Enterobacter hormaechei* subsp., *Halomonas* sp., *Hyphomicrobium* sp., *Ochrobactrum anthropi, Rhodobacter* sp.*, Photorhabdus luminescens, Planococcus* sp., *Pseudoalteromonas* sp., *Psychrobacter glacincola, Psychrobacter* sp. *Roseavarius crassostreae, Staphylococcus* sp., *Steigerwaltii* sp., uncultured Arctic sea ice bacterium, uncultured soil bacterium, uncultured beta-proteobacterium, uncultured gram-positive bacterium	[7,8,13]
Sponge *Mycale adhaerens*	*Alteromonas alvinellae, Kocuria rhizophila, Mesophilum* sp., *Microbulbifer hydrolyticus, Micrococcus kristinae, Pseudoalteromonas piscicida, Pseudoalteromonas spongiae, Shewanella alga, Staphylococcus cohnii, Tenacibaculum* sp., *Vibrio fluvialis, Vibrio furnissii, Vibrio halioticoli, Vibrio nereis*	[14]
Sponge *Callyspongia* sp.	*Alteromonas macleodii, Alteromonas marina, Bacillus cereus, Bacillus hwajinpoensis, Bacillus megaterium, Bacillus thuringiensis serovar konkukian, Erythrobacter flavus, Exiguobacterium gaetbuli, Exiguobacterium marinum, Marinobacter aquaeolei, Micrococcus luteus, Planococcus citreus, Pseudovibrio denitrificans, Psychrobacter* sp., *Ruegeria atlantica, Silicibacter lacuscaerulensis, Vibrio aestuarianus, Vibrio corallilyticus, Vibrio fischeri, Vibrio harveyi, Vibrio hollisae, Vibrio natriegens, Vibrio tubiashi, Vibrio probioticus*	[15]
Sponge *Hymeniacidon perleve* Coral *Eupexaura curvata* Seaweeds *Gymnogongrus flabelliformis, Laurencia flabelliformis, Laminaria japonica, Lomentaria catenata, Sargassum thunbergii, Ulvafasciata, Ulvapertusa*	*Alteromonas* sp, *Bacillus* sp., *Flavobacterium* sp., *Pseudomonas* sp.	[16]
Soft coral *Dendronephthya* sp.	*Flexibacter* sp., *Pseudoalteromonas* sp., *Tenacibaculum mesophilum, Vibrio ichthyoenteri*	[17,18]
	**Archaea**	
Sponge *Pachychalina* sp.	Archaeal clone	[19]
Sponge *Theonella swinhoei*	*Cenarchaeum*	Data to be published
	**Fungi**	
Sponge *Phakellia fusca*	*Ascomycota sp., Aspergillns candidus, Aspergillus fiimigatus, Aspergillus ochraceus, Apiospora montagnei, Candi da parapsilosis, Cladosporium* sp., *Davidiella tassiana, Didymocrea sa dasivanii, Fusarium* sp., *Hypo crea koningii, Lentomitella cir rhosa, Marasmius alii aceus, Nigr ospora or yzae, P aecilomyces li lacinus, Penicillium chrysogenum, Penicillium purpurogenum, Pestalotiopsis guepinii, Scopu lariopsis brevicaulis, Rhizomucor pusillus*	Data to be published
Sponge *Theonella swinhoei*	*Ascomycota* sp., *Aspergillus versicolor, Davidiella tassiana, Fusariu m* sp., *Paecilomyces lilacinus, Penicillium chrysogenum, Penicillium pinophilum*	Data to be published

**Table 2:** Pharmaceutical metabolites from marine microbial symbionts in the China Sea

Host	Microorganism	Compound	Bioactivity	Ref.
Sponge *Hymeniacidon perleve*	*Pseudoalteromonas piscicida*	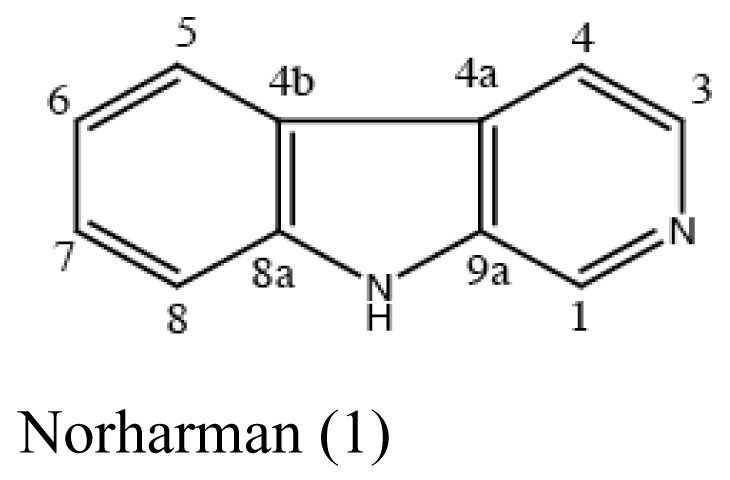	Antimicrobial activity	[20]
Sponge *Stelletta tenuis*	*Alcaligenes faecalis* A72	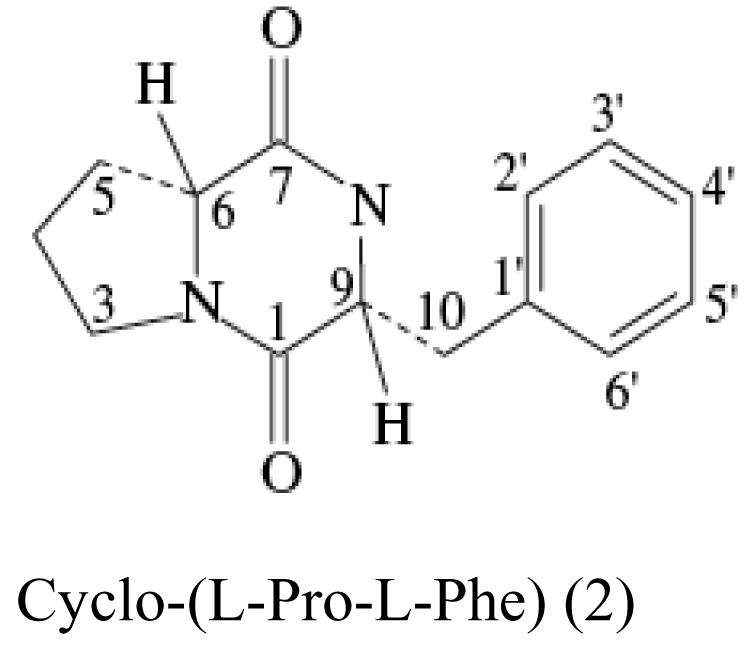	Moderate antimicrobial activity	[21]
Sponge *Mycale plumose*	*Saccharopolyspora* sp. nov.	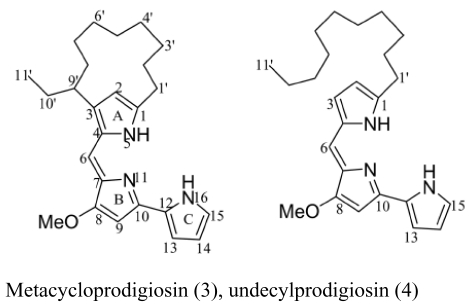	Cytotoxic activity	[22]
Sponge *Dysidea avara*	*Bacillus vallismortis* C89	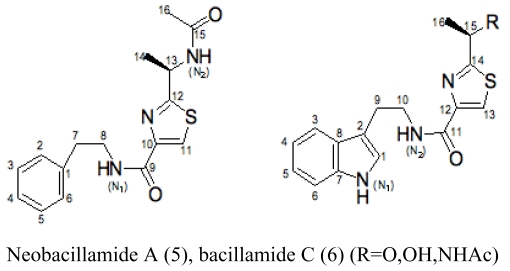	Red-tide algae inhibited	[23,24]
Sponge *Mycale plumose*	*Penicillium auratiogriseum*	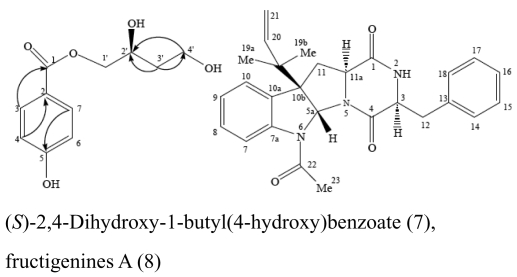	Cytotoxic activity	[25]
Sponge *Axinella* sp.	*Myrothecium* sp.	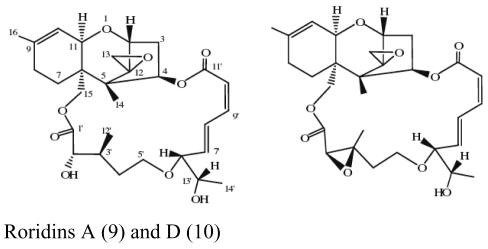	Antifungal activity	[26]
Sponge *Acanthella cavernosa*	*Letendraea helminthicola*	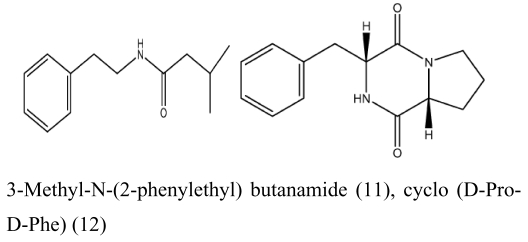	Antifouling	[27]
Sponge *Mycale plumose*	*Penicillium aurantiogriseum SP0-19*	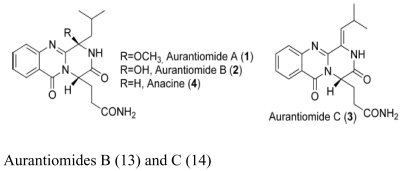	Moderate cytotoxic activity	[28]
Sea squirt (unidentified)	*Penicillium stoloniferum* QY2-10	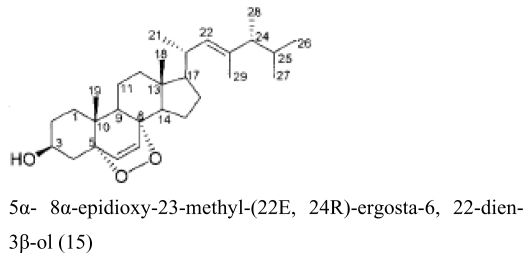	Cytotoxic activity	[29]
Marine *Bugula* sp.	*Rhizopus* sp.	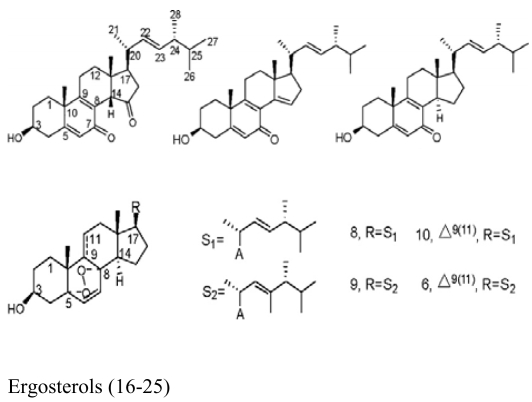	Cytotoxic activity	[30]
Holothurian	*Aspergillus fumigatus*	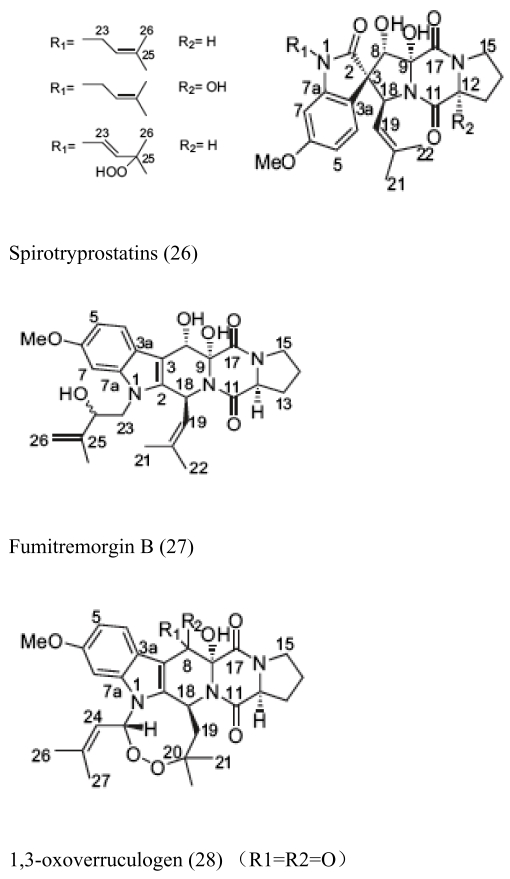	Cytotoxic activity	[31]
Brown alga *Sargassum* sp.	Unidentified endophytic fungus No. ZZF36	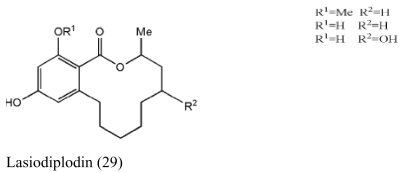	Antimicrobial activity	[32]
Brown alga *Colpomenia sinuosa*	*Aspergillus niger EN-13*	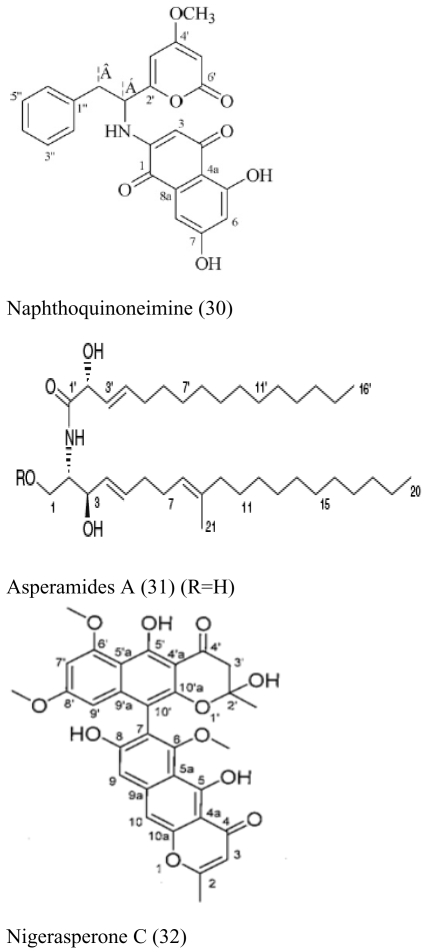	Moderate antifungal activity	[33,34,35]
Red alga *Polysiphonia urceolata*	*Chaetomium globosum*	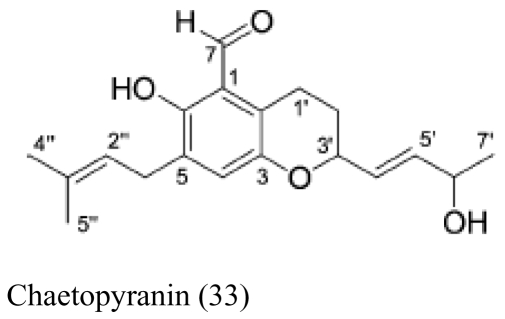	Moderate to weak cytotoxic activity	[36]
